# Habitat influences skeletal morphology and density in the snailfishes (family Liparidae)

**DOI:** 10.1186/s12983-021-00399-9

**Published:** 2021-04-16

**Authors:** M. E. Gerringer, A. S. Dias, A. A. von Hagel, J. W. Orr, A. P. Summers, S. Farina

**Affiliations:** 1 State University of New York at Geneseo, Geneseo, NY 14454 USA; 2 Whitman College, Walla Walla, WA 99362 USA; 3 University of Washington, Seattle, WA 98195 USA; 4 Alaska Fisheries Science Center, RACE Division, NOAA Fisheries, Seattle, WA 98115 USA; 5 Friday Harbor Labs, Biology and SAFS, University of Washington, Friday Harbor, WA 98250 USA; 6 Howard University, Washington, DC 20059 USA

**Keywords:** Mineralization, Buoyancy, Hydrostatic pressure, Micro-CT, Bone density, Deep sea

## Abstract

We tested the hypothesis that deep-sea fishes have poorly mineralized bone relative to shallower-dwelling species using data from a single family that spans a large depth range. The family Liparidae (snailfishes, Cottiformes) has representatives across the entire habitable depth range for bony fishes (0 m–> 8000 m), making them an ideal model for studying depth-related trends in a confined phylogeny. We used micro-computed tomography (micro-CT) scanning to test three aspects of skeletal reduction in snailfishes (50 species) across a full range of habitat depths: 1) reduction of structural dimensions, 2) loss of skeletal elements, and 3) reduction in bone density. Using depth data from the literature, we found that with increasing depth, the length of the dentary, neurocranium, and suborbital bones decreases. The ventral suction disk decreases width with increasing maximum habitat depth and is lost entirely in some deeper-living taxa, though not all. Although visual declines in bone density in deeper-living taxa were evident across full skeletons, individual densities of the lower jaw, vertebra, suction disk, hypural plate, and otoliths did not significantly decline with any depth metric. However, pelagic and polar taxa tended to show lower density bones compared to other species in the family. We propose that skeletal reductions allow snailfishes to maintain neutral buoyancy at great depths in the water column, while supporting efficient feeding and locomotion strategies. These findings suggest that changes in skeletal structure are non-linear and are driven not only by hydrostatic pressure, but by other environmental factors and by evolutionary ancestry, calling the existing paradigm into question.

**Supplementary Information:**

The online version contains supplementary material available at 10.1186/s12983-021-00399-9.

## Background

The majority of the habitable biosphere on Earth is in the deep sea, characterized by high hydrostatic pressures, cold temperatures, and the absence of sunlight (reviewed by [92]). Deep-sea habitats vary—from cold-water corals to abyssal plains, hydrothermal vents to deep-sea trenches, mesopelagic open waters to submarine canyons—and each are accompanied by distinct conditions and associated fauna. Specific environmental conditions such as temperature, hydrostatic pressure, and nutrient availability have given rise to a wide array of adaptations in deep-sea organisms. As a result, deep-sea fishes are incredibly diverse (e.g. [45, 90]), with specialized traits to survive, feed, reproduce, and hide in deep-sea habitats (e.g. [21, 51, 90, 93]).

Hydrostatic pressures in the deep sea reach more than one-thousand times atmospheric pressure, up to 100 MPa, posing an evolutionary challenge for fishes to radiate into the deep oceans. Fishes have evolved mechanisms to cope with these high hydrostatic pressures on multiple scales, from the molecular to the organismal (e.g. [101, 110]). Many deep-sea organisms, including fishes, use pressure adaptations such as increased levels of polyunsaturated fatty acids that maintain fluidity of cellular membranes under high pressures and low temperatures [3, 15, 98], specialized enzymes and proteins [39, 42, 97, 102], and protein-stabilizing molecules called piezolytes that prevent water from being pushed into the active sites of enzymes and proteins (reviewed by [113]). One notable example is the protein-stabilizing osmolyte trimethylamine-N-oxide (TMAO) that accumulates in fish cells with increasing habitat depth. In hadal trenches, at around 8200 m, cells become isosmotic with seawater due to these high TMAO concentrations, resulting in a likely physiological constraint on maximum depth for fishes [111].

High hydrostatic pressures pose an additional challenge for fishes—to maintain neutral buoyancy. The bony skeleton of fishes provides support, protection, and attachment surfaces for muscles. However, a skeleton is dense and reduces buoyancy. Gas bladders for buoyancy become increasingly difficult to inflate with increasing habitat depth [89, 96]. Some deep-sea species use a subdermal gelatinous tissue to maintain neutral buoyancy without a gas-filled bladder [28, 40, 112], while others have lipid-filled swim bladders (e.g. [43, 71]) and even lipid-rich bones [59] to add positive buoyancy. Deep-sea anglerfishes, gulper eels and several other lineages have poorly mineralized bone, which is assumed to be an adaptation to maintain neutral buoyancy by reducing the weight of the body’s densest tissues [12, 14, 19, 45, 57, 93]. Decreased mineralization of the skeleton can be achieved by reducing the size and thickness of bones, or by reducing the amount of mineral (such as calcium, phosphate, or sulfate) in the bone [93].

The diversity of deep-dwelling fishes (e.g. [90]), makes it difficult to distinguish specific deep-sea adaptations from variations across phylogeny or from other factors. This study examines skeletal properties across a depth range from a single family, the snailfishes (Liparidae). Liparids are generally small (< 20 cm), tadpole-shaped fishes that lack scales. Most liparids have a ventral sucking disk, used to stick to rocks and other substrates in shallow environments (e.g. [7]). In some deep-water species, the disk may be used in carcinophyly, a parasitic reproductive strategy where the fish attaches to the carapace of a king crab [114] and deposits its eggs inside the carapace on the gills [35, 106]. The Liparidae are a morphologically distinct and monophyletic family [69, 82, 99] with over 430 described species [32]. Snailfishes live in cold polar to temperate waters and have the largest bathymetric range of any fish family—from the intertidal to over 8000 m [10, 34, 61, 72]. As such, the liparids represent an appropriate system to investigate adaptation into the deep sea within a constrained phylogeny. Deep-sea liparids have been described as having “less firm” bodies and weakened skeletons in comparison to their intertidal counterparts, so there is anecdotal evidence of skeletal variation [7]. Phylogenetic evidence suggests that liparids evolved from a shallow-living ancestor, following which, the majority of the family diversified from a deep-water clade with both demersal and pelagic lifestyles [82]. A trend of evolutionary reduction in certain morphological features (pelvic fins, pseudobranchial filaments, skin spinules, brain mass, visual system) in liparid species more specialized for deep-sea habitats has also been noted [55, 58, 64].

Here, we investigate changes in skeletal density and form with habitat across the family Liparidae through micro-computed tomography (micro-CT), covering the complete bathymetric range of bony fishes. We predict that bones with fewer functional demands at depth will be reduced, while other crucial bones will remain relatively large and dense, even at great depths. We test three hypotheses related to skeletal declines: 1) dimensions of specific bones become reduced with increasing habitat depth; 2) skeletal elements that are not adaptive in a deep-sea habitats are lost in deep-sea taxa; and 3) density of individual bone structures declines with increasing depth. We then qualitatively examine other habitat-related trends in skeletal density such as between pelagic and demersal taxa and polar and subpolar species.

**Fig. 1:**
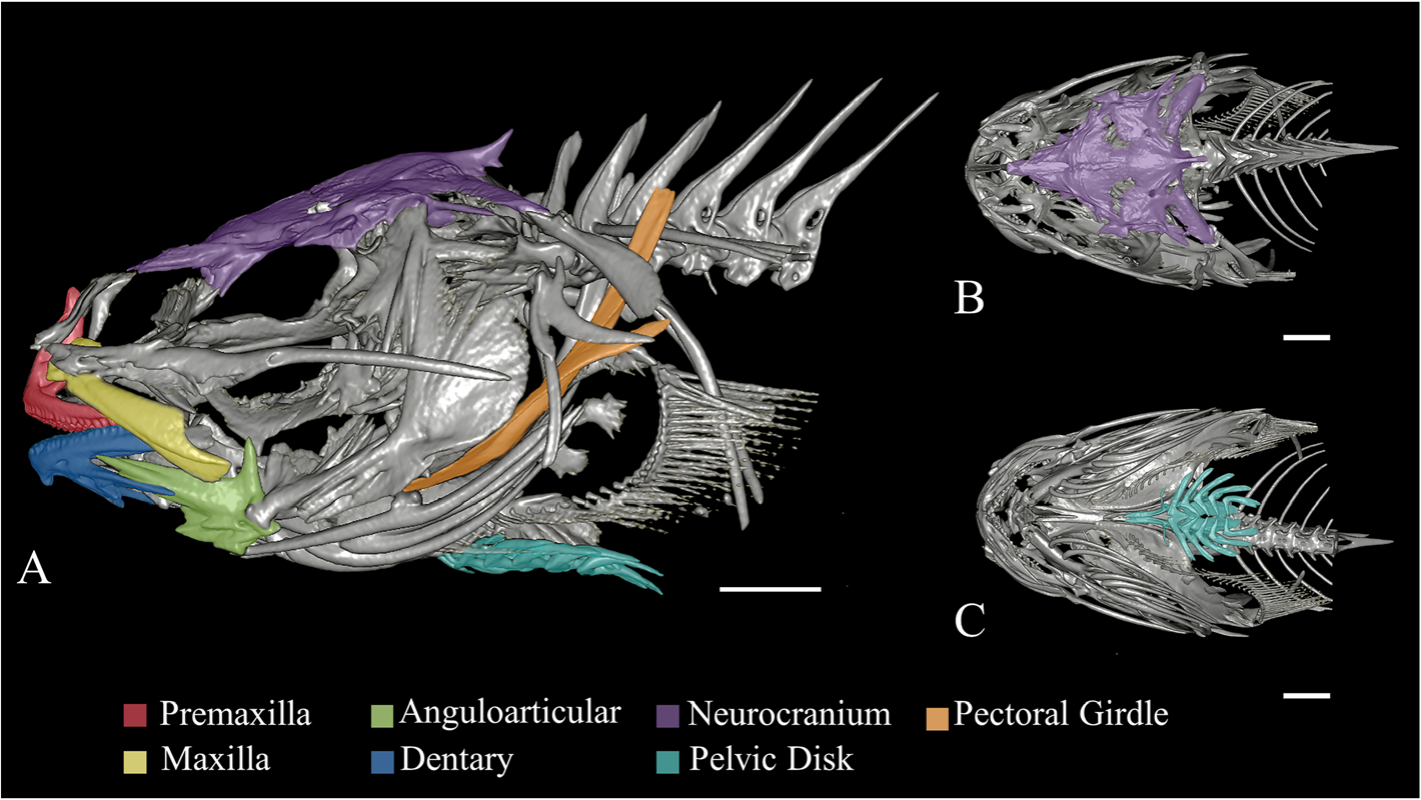
Skeletal elements measured for linear morphometrics. **a** Lateral view, **b** dorsal view, **c** ventral view of *Liparis florae*, an intertidal snailfish (UW 040065). Bones measured in this study are highlighted in the following colors: premaxilla = red, anguloarticular = green, neurocranium = purple, pectoral girdle = orange, maxilla = yellow, dentary = blue, pelvic disk = teal. Scale bars are 0.25 cm

To test the first hypothesis—that bone dimensions would be reduced with increasing depth—we measured three areas of the skeleton: the jaw, pectoral girdle, and neurocranium (specific bones are shown in Fig. 1). We expected the neurocranium would be most reduced, because selective pressure for protection of the brain may be diminished due to decreased predation risk at increased depths [46]. We predicted the bones of the pectoral girdle and jaws would remain at least as large in deep-water fishes as in nearshore relatives due to their key role in suction-feeding and swimming. Snailfishes from both intertidal waters and hadal trenches have been observed using pectoral fin motion to navigate and obtain resources (e.g. [40, 53, 107]). Snailfishes are primarily suction feeders (e.g. [2]), relying on the bones of the jaw to rapidly protrude and contribute to negative pressures in the mouth to capture prey [48, 62, 109]. Compared to other co-occurring fishes, some Antarctic liparids have a lower mechanical advantage and bite force but a higher suction index [2]. Both intertidal and deep-sea species rely on this suction feeding strategy to catch small crustaceans—their main prey [16, 41, 56, 73].

Skeletons in deep-water fishes could also be reduced through the complete loss of bone structures. We hypothesize that certain skeletal elements are lost with increasing depth, due to changing evolutionary drivers. While snailfishes at any habitat depth would require certain bones, for example, pharyngeal jaws, other skeletal elements could be less important to life in the deep sea. For example, the ventral suction disk might be less important in deep-sea habitats, where hydrodynamic forces due to wave action are not a factor in evolutionary success.

**Fig. 2:**
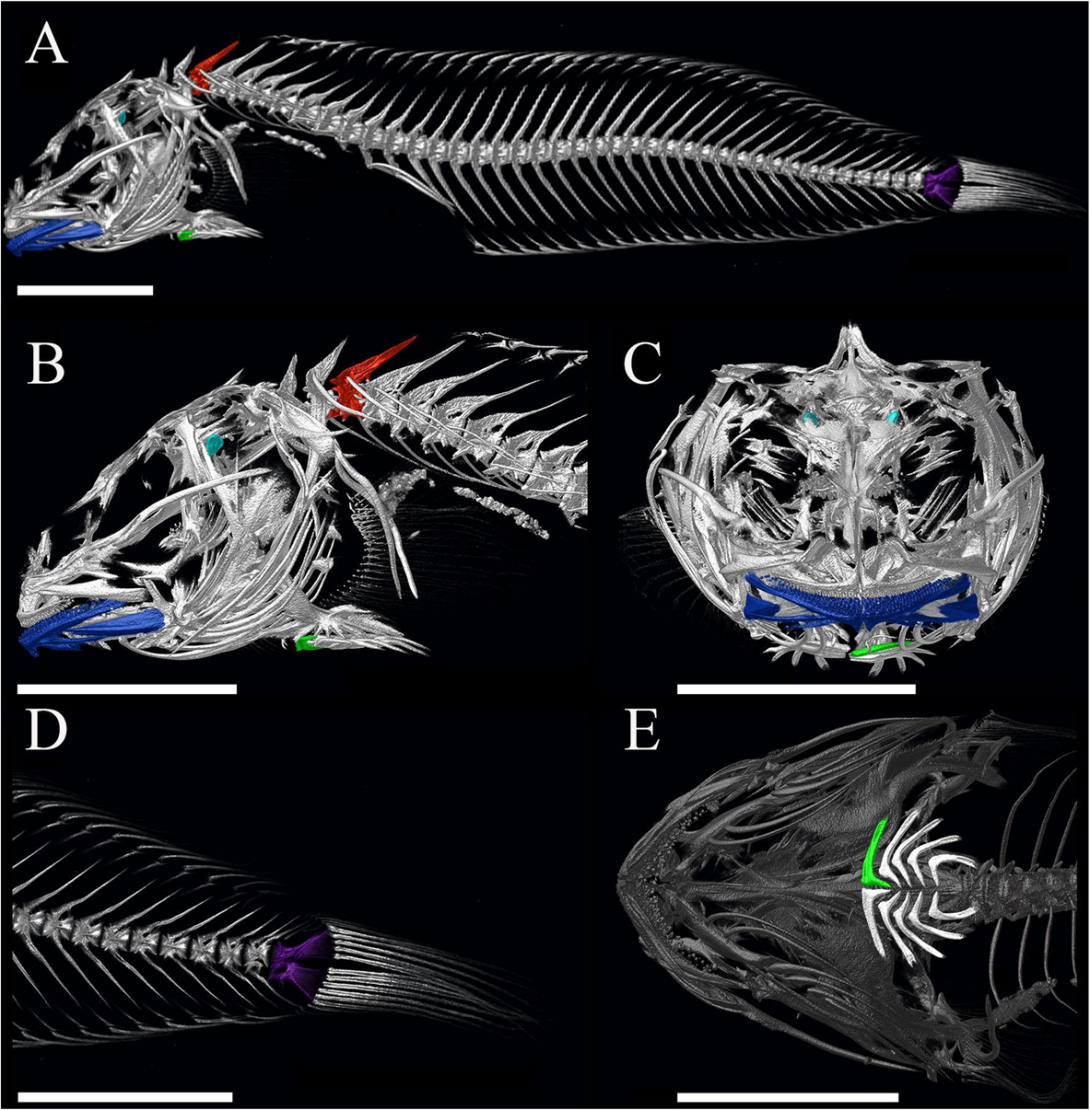
Segmentations showing analyzed bones for one shallow-living, dense species: *Liparis miostomus* (UW 041391). **a** Full body image highlighting all analyzed bones: third vertebra = red, lower jaw = dark blue, first left disk pterygiophore = green, hypural plate = purple, sagittal otoliths = light blue **b** lower jaw, sagittal otoliths, third vertebra, and first left disk pterygiophore, **c** jaw measurement includes the dentary and anguloarticular bone but does not include the joint, **d** hypural plate, and **e** ventral view of full suction disk showing highlighted first left disk pterygiophore. Scale bars are 1 cm

A decline in bone density with increasing depth in deep-sea fishes is likely complicated by interactions with bone function. Bone mineralization is related to stiffness and strength, which in turn can limit performance. We quantified density in five bones with different biological functions and consequent performance requirements: the lower jaw, the pelvic suction disk, a vertebra, the hypural plate, and the sagittal otoliths (Fig. 2). Density of the lower jaw was measured as an indication of the energy the fish has devoted to mineralizing feeding structures. We expected no effect of depth on jaw density as liparids rely on a diet of small crustaceans across a broad range of habitat depths [41] and deep-living snailfishes should have similar requirements in feeding morphology to their shallow-living counterparts. We measured the first left pelvic disk pterygiophore as a proxy for suction disk performance with increasing depth. Here, we expected to see a reduction in density as deep-sea habitats are not subject to wave action and high current flow. The hypural plate captures the undulatory locomotor performance, with the hypothesis that fishes in deeper environments would have limited need for long burst swimming due to decreased interaction distances between predator and prey with declining light levels [11, 24]. We measured the density of the third vertebra, which we predicted to reflect the overall density of the specimen and give insights into both locomotion and buoyancy. Reduced vertebral density with increasing habitat depth could also signal the need to maintain neutral buoyancy under high pressures. The sagittal otoliths, calcium carbonate structures in the inner ear that function in hearing, were included because their density reflects a sensory need that should not change with increasing depth and so might remain unchanged with depth. We then explore patterns of bone density with other habitat parameters, including latitude and lifestyle.

## Results

**Fig. 3:**
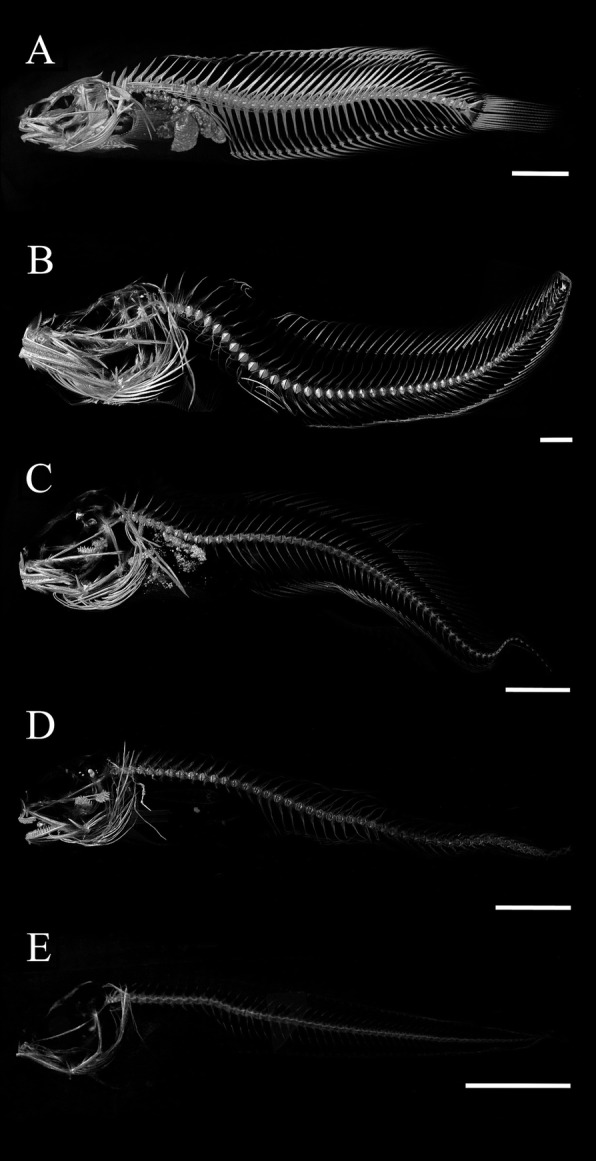
Diversity of the snailfishes in micro-CT. Sample scans across the major snailfish genera: **a** *Liparis florae*, tidepool snailfish, 0 m collection depth, 87.7 mm SL. **b** *Careproctus ovigerus*, abyssal snailfish, 1109 m collection depth, 190.5 mm SL. **c** *Paraliparis grandis*, grand snailfish, 834 m collection depth, 102.3 mm SL. **d** *Pseudoliparis swirei*, Mariana snailfish, 7949 m collection depth, 104 mm SL, holotype. **e** *Nectoliparis pelagicus*, tadpole snailfish, 392 m collection depth, 54.2 mm SL. Brightness has been optimized for each scan to show structure and density is not comparable across these images. Scale bars are 1 cm

We scanned 50 species of snailfishes (Family Liparidae) in 13 genera across the complete habitat depth range for bony fishes (0–8000 m). Specimens from different genera varied in scan brightness and morphology across taxa (Fig. 3). These CT scans were used to explore three potential skeletal declines across varying habitats: dimensional reductions, loss of skeletal elements, and reductions in bone density.

### Bone dimension reduction

**Fig. 4:**
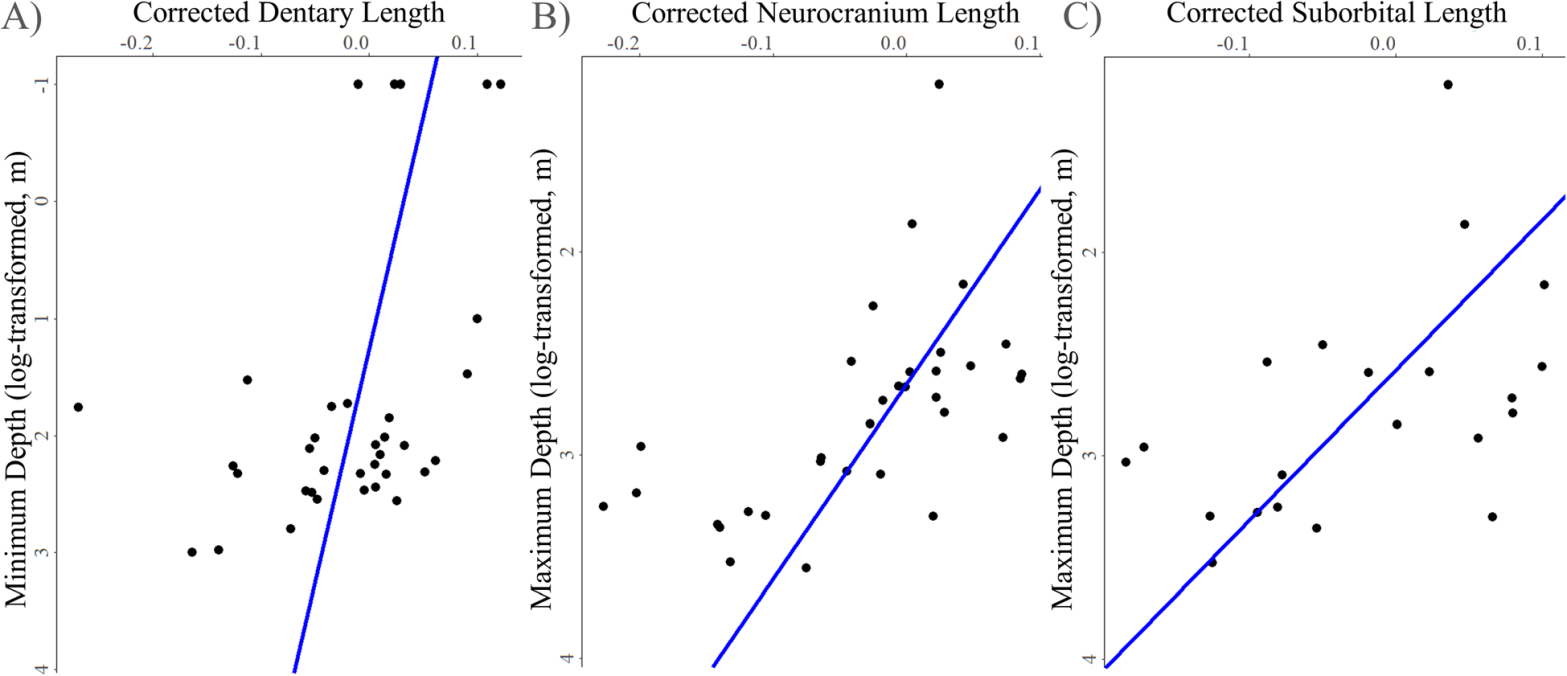
Certain skeletal bone dimensions decline with increasing habitat depth in snailfishes. Select results from PGLS modeling are shown for **a** the dorsal fork length of the dentary (dfl) with minimum depth, **b** neurocranium length with maximum depth, and **c** suborbital length with maximum depth. PGLS fits are shown in blue. Depths are log-transformed. Linear measurements have been log-transformed and corrected to both specimen standard length and phylogenetic position. See text for details. Significance tests for each PGLS model are shown in Table 1

Length and width of skeletal elements of the jaw, neurocranium, pectoral girdle, and suction disk were measured for 35 species from 12 genera (**Supplementary Table** **1**). Correlations of phylogenetic independent contrasts showed that there was a decrease in some bone lengths with increasing depth, while other bone lengths did not differ significantly along a depth gradient (Table 1). The relative length of the dentary (dorsal fork and ventral fork lengths) significantly decreased with increasing minimum depth (Fig. 4**a,** dorsal fork length: α = 0.05, df = 35, *p* = 0.0432; ventral fork length: α = 0.1, df = 35, *p* = 0.0707). Dentary lengths did not significantly decrease with collection or maximum depth (Table 1). The maxilla (length and width), premaxilla (lateral length and ascending process length), and anguloarticular (length) were not significantly related to habitat depth by any metric (Table 1). Neurocranium length decreased with maximum depth (α = 0.05, df = 34, *p* = 0.0007; Fig. 4**b**) and collection depth (α = 0.1, df = 35, *p* = 0.0760). The length of the suborbital bone significantly decreased with increasing habitat depth by all metrics (Fig. 4**c;** α = 0.05, minimum depth: df = 22, *p* = 0.0010: maximum depth: df = 21, *p* = 0.0002; collection depth: df = 22, *p* = 0.0002), while suborbital width was not related to depth (Table 1). Pectoral girdle length did not significantly correlate with any depth metric after phylogenetic correction (Table 1). Maximum disk width decreased with increasing maximum depth (α = 0.1, df = 21, *p* = 0.0572), but did not relate to minimum or collection depth (Table 1). AIC values for PGLS models suggested that minimum depth often provided the best fit metric (maxilla length and width, premaxilla ascending process length, lengths of both the dorsal and ventral forks of the dentary, and anguloarticular length), though for other bones maximum depth (premaxilla length, neurocranium length, and maximum disk width) and collection depth (suborbital length and pectoral girdle length) resulted in the lowest AIC values (Table 1). Standard-length corrected phylogenetic residuals correlated with habitat depth across most bones and depth metrics (ANOVA, *p* < 0.05 for maxilla width, the ascending process of the premaxilla, premaxilla length, dorsal fork length of the dentary, neurocranium length, and suborbital length for all depth metrics, **Supplementary Table** **2**).

**Table 1:** The relationship between depth and skeletal element dimension according to PGLS significance tests

Linear Measurement	Minimum Depth				Maximum Depth				Collection Depth			
	*df*	*Coefficient*	*p-value*	*AIC*	*df*	*Coefficient*	*p-value*	*AIC*	*df*	*Coefficient*	*p-value*	*AIC*
Maxilla Length	*35*	-0.0172885	0.2025	**-58.78**	*34*	0.0222065	0.4061	-55.11	*35*	0.0200694	0.2731	-58.32
Maxilla Width	*35*	-0.0511695	0.1018	**-0.898**	*34*	-0.0106798	0.8589	0.302	*35*	0.0088989	0.8358	1.939
Premaxilla Ascending Process	*35*	-0.0222041	0.1755	**-45.66**	*34*	-0.0264503	0.3911	-45.40	*35*	-0.0048768	0.8273	-43.74
Premaxilla Length	*35*	-0.0085254	0.5687	-51.25	*34*	0.0427720	0.1133	**-55.37**	*35*	0.0252946	0.2043	-52.63
Dentary (dfl)	*35*	-0.0251871	**0.0432	**-66.08**	*34*	0.0162499	0.5072	-60.88	*35*	0.0122456	0.4772	-62.22
Dentary (vfl)	*35*	-0.0351275	*0.0707	**-34.50**	*34*	-0.0247770	0.5171	-30.60	*35*	-0.0138015	0.6060	-31.27
Anguloarticular Length	*35*	-0.0172143	0.2267	**-55.30**	*34*	-0.0044229	0.8711	-53.40	*35*	0.0088911	0.6458	-53.96
Neurocranium Length	*35*	-0.0197188	0.2555	-41.42	*34*	-0.1045617	**0.0007	**-51.15**	*35*	-0.0408159	*0.0760	-43.41
Suborbital Length	*22*	-0.0619564	**0.0010	-23.56	*21*	-0.1357821	**0.0002	-25.07	*22*	-0.0799057	**0.0002	**-27.42**
Suborbital Width	*35*	-0.0141958	0.3037	**-57.27**	*34*	0.0321298	0.2320	-55.12	*35*	0.0099389	0.5947	-56.44
Pectoral Girdle Length	*35*	-0.0024060	0.8177	-76.27	*34*	-0.0115989	0.5655	-74.01	*35*	0.0099849	0.4756	**-76.76**
Maximum Disk Width	*21*	-0.0025815	0.9245	-8.805	*21*	-0.0927278	*0.0572	**-12.90**	*21*	-0.0250137	0.4963	-9.320

Specimen collection depth, species minimum depth, and species maximum depth (in meters) were tested independently. Dentary linear measurements are shown for dorsal fork length (dfl) and ventral fork length (vfl). Total degrees of freedom (df) for each test are shown. Significant *p*-values below an alpha threshold of 0.05 are marked with two asterisks and bold type, those below the 0.1 alpha threshold are marked with a single asterisk and bold type. Estimated regression coefficient and Akaike information criteria (AIC) are reported for each model. The depth metric with the lowest AIC value for each bone appears in bold type

### Loss of skeletal elements

**Fig. 5:**
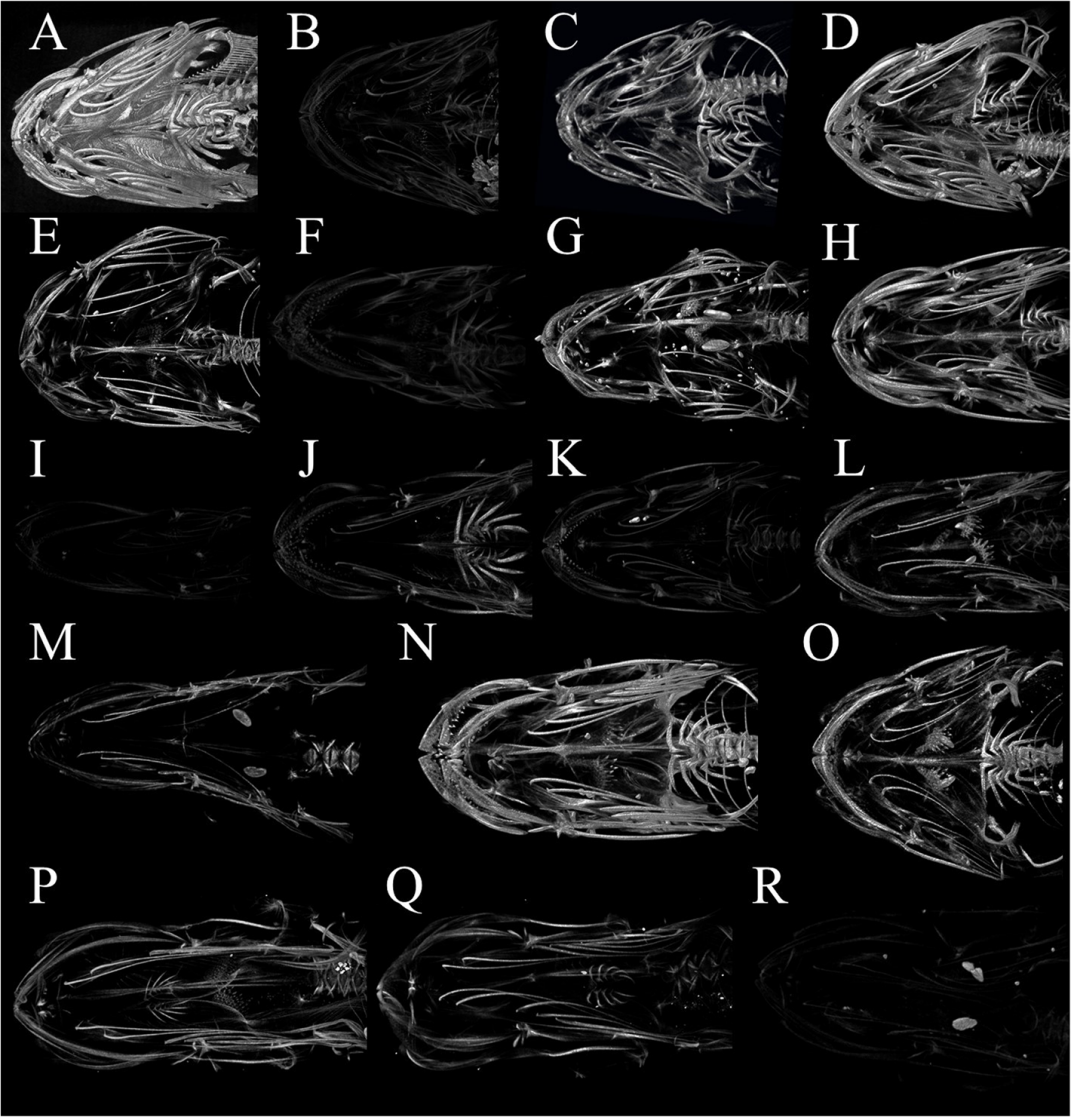
Ventral views of a select 18 species of snailfishes, showing variation of the pelvic suction disk (labeled in Figs. 1 and 2). Scans are ordered by maximum habitat depth, from shallowest to deepest. Images are not to scale, see disk lengths below for relative sizes. Brightness is standardized across scans and is indicative of specimen density. **a ***Liparis florae*, maximum depth 15 m, demersal, temperate, disk length 6.26 mm, **b ***Liparis greeni*, maximum depth 21 m, demersal, temperate, **c ***Liparis rutteri*, maximum depth 73 m, demersal, temperate, disk length 5.75 mm, **d ***Liparis bristolensis*, maximum depth 144 m, demersal, temperate, disk length 5.73 mm, **e** *Liparis pulchellus*, maximum depth 183 m, demersal, mid-latitude, **f ***Crystallichthys cyclospilus*, maximum depth 312 m, demersal, temperate, disk length 9.38 mm, **g ***Liparis tessellatus*, maximum depth 346 m, demersal, temperate, disk length 5.55 mm, **h ***Liparis fucensis*, maximum depth 388 m, demersal, temperate, disk length 3.74 mm, **i** *Careproctus comus*, maximum depth 400 m, demersal, temperate, disk length 2.58 mm, **j ***Temnocora candida*, maximum depth 400 m, demersal, temperate, **k ***Allocareproctus unangas*, maximum depth 465 m, demersal, temperate, disk length 6.08 mm, **l ***Liparis fabricii*, maximum depth 520 m, sometimes pelagic, polar, disk length 5.80 mm, **m ***Careproctus acanthodes*, maximum depth 582 m, demersal, temperate, **n ***Allocareproctus tanix*, maximum depth 620 m, demersal, temperate, disk length 5.24 mm, **o ***Liparis tunicatus*, maximum depth 620 m, demersal, polar, disk length 6.93 mm, **p ***Careproctus furcellus*, maximum depth 1270 m, demersal, temperate, **q ***Careproctus colletti*, maximum depth 1556 m, demersal, temperate, **r ***Careproctus cypselurus*, maximum depth 1993 m, demersal, temperate, disk length 2.28 mm. Each specimen shown has a ventral suction disk, though some are greatly reduced in size and density

Each snailfish scan was analyzed for the presence of major skeletal features. Although declining density made elements such as pectoral fin rays, pectoral radials, and caudal vertebrae more difficult to see in the micro-CT scans, the only major skeletal element that was absent in some taxa was the pelvic suction disk. The disk was absent in 15 of the 45 species analyzed (Table 2). The genera that lacked disks were *Acantholiparis*, *Elassodiscus, Lipariscus, Nectoliparis, Paraliparis,* and *Rhinoliparis*. The disk in *Elassodiscus* is reduced to small nonfunctional lobes, a condition we considered as absent here. Examples of variation in disk size, shape and development are shown in Fig. 5. All members of the polyphyletic genus *Careproctus* have suction disks, yet the distinct but tiny disk of *Careproctus ostentum* was not visible in the scanned specimen. Due to this discrepancy, this species was not included in our ancestral state reconstructions.

**Table 2:** Disk status in 45 species of snailfishes visualized in micro-CT

Species	Collection Depth (m)	Minimum Depth (m)	Maximum Depth (m)	Disk Present?
*Acantholiparis opercularis*	1000	300	3609	No
*Allocareproctus kallaion*	441	278	458	Yes
*Allocareproctus tanix*	158	104	620	Yes
*Allocareproctus unangas*	387	176	465	Yes
*Careproctus acanthodes*	150	114	582	Yes
*Careproctus bowersianus*	848	629	1032	Yes
*Careproctus colletti*	994	200	1556	Yes
*Careproctus comus*	303	146	400	Yes
*Careproctus cypselurus*	1017	214	1993	Yes
*Careproctus faunus*	348	120	422	Yes
*Careproctus furcellus*	933	98	1270	Yes
*Careproctus longifilis*	–	1900	3499	Yes
*Careproctus ostentum*	332	165	700	No*
*Careproctus ovigerus*	1109	1109	2910	Yes
*Careproctus phasma*	76	57	184	Yes
*Careproctus scottae*	203	71	390	Yes
*Careproctus simus*	455	213	819	Yes
*Careproctus staufferi*	256	205	366	Yes
*Crystallichthys cyclospilus*	120	54	312	Yes
*Elassodiscus nyctereutes*	616	362	1200	No
*Elassodiscus tremebundus*	700	130	1248	No
*Liparis bristolensis*	10	10	144	Yes
*Liparis fabricii*	28	0	520	Yes
*Liparis florae*	0	0	15	Yes
*Liparis fucensis*	45	0	388	Yes
*Liparis gibbus*	38	30	540	Yes
*Liparis greeni*	0	0	21	Yes
*Liparis pulchellus*	33	9	183	Yes
*Liparis rutteri*	38	0	73	Yes
*Liparis tessellatus*	34	34	346	Yes
*Liparis tunicatus*	43.8	0	620	Yes
*Lipariscus nanus*	477	58	910	No
*Lopholiparis flerxi*	278	121	285	Yes
*Nectoliparis pelagicus*	392	200	–	No
*Paraliparis cephalus*	622	294	1799	No
*Paraliparis dactylosus*	869	212	1073	No
*Paraliparis grandis*	834	105	1995	No
*Paraliparis holomelas*	188	55	2972	No
*Paraliparis paucidens*	1018	950	2275	No
*Paraliparis pectoralis*	950	308	1536	No
*Paraliparis rosaceus*	999	999	3358	No
*Paraliparis ulochir*	1018	182	1900	No
*Pseudoliparis swirei*	7949	6198	8098	Yes
*Rhinoliparis attenuatus*	1018	350	2189	No
*Temnocora candida*	238	64	400	Yes

Minimum and maximum depth are provided for reference. *The externally visible suction disk of *Careproctus ostentum* was not evident in the scan. Depth sources are shown in Table 4

**Fig. 6:**
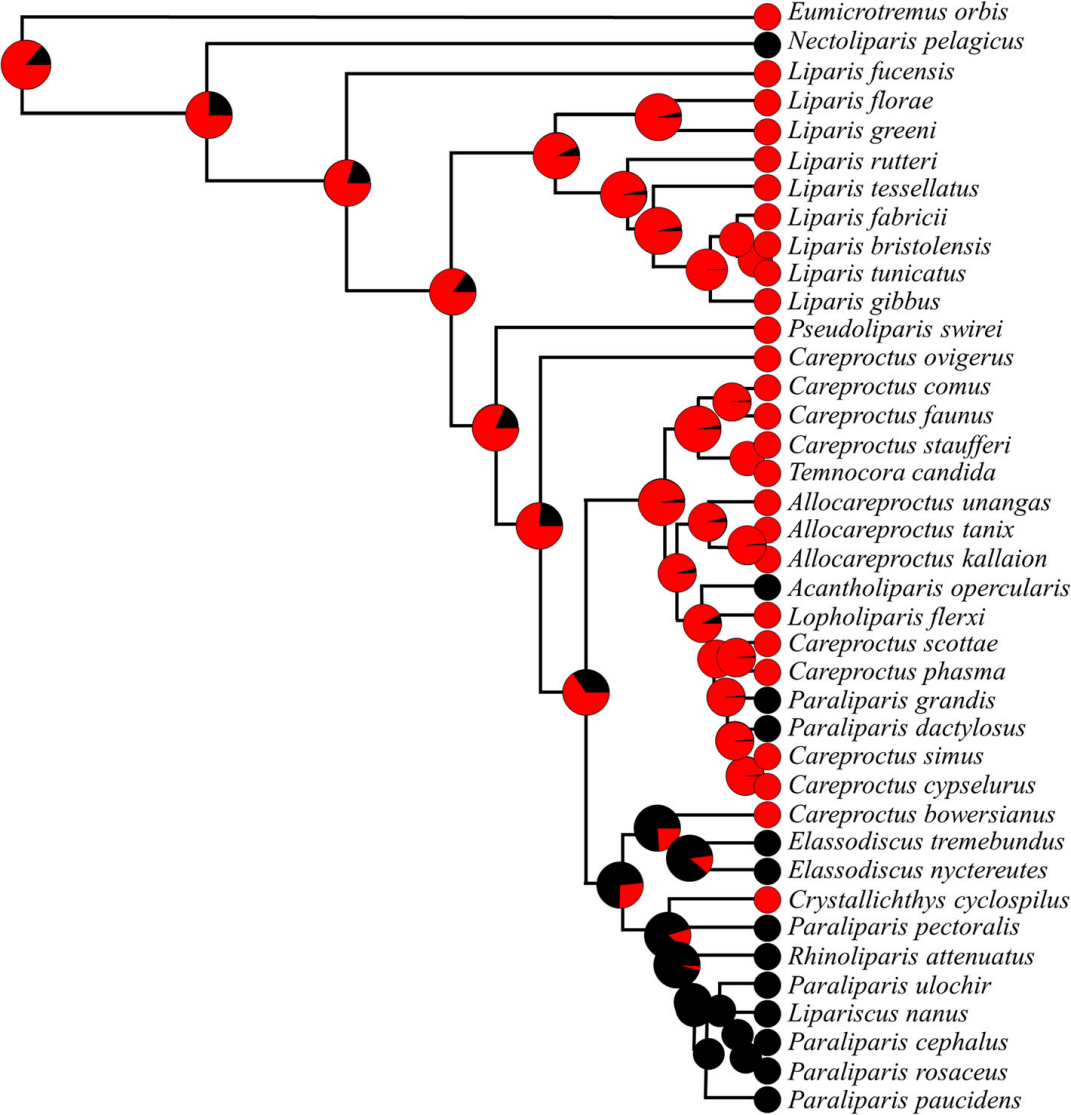
Ancestral state reconstruction predicting presence of the ventral suction disk across the evolutionary history of the family Liparidae. At the terminal nodes, species with a disk are marked in red, while those without are marked in black. Ancestral nodes illustrate the percentage of simulations that were predicted to have a suction disk (red = disk present, black = disk absent, *n* = 1000 simulations). The outgroup is the Pacific spiny lumpsucker, *Eumicrotremus orbis* (Family Cyclopteridae), which has a prominent and highly functional ventral suction disk (e.g. [1])

We examined the presence or absence of a ventral suction disk in relation to phylogeny and habitat depth. With the *phytools* package [94] in the statistical programming platform R [91], we found marginal ancestral state estimates and used the phylANOVA function to test for an effect of depth in predicting structure loss. Maximum habitat depth was a significant predictor of disk absence, while collection depth and minimum depth were not (phylANOVA, nsim = 1000, depths log-transformed; collection depth F = 8.364424, *p* = 0.146; maximum depth F = 14.735598, *p* = 0.038; minimum depth: F = 5.950088, *p* = 0.217). We then simulated ancestral states of disk presence or absence using stochastic character mapping in the simmap function [4] based on the tree of Orr et al. [82]. Our ancestral state reconstruction for the presence of the ventral suction disk across evolutionary time is shown in Fig. 6. These data indicate that the ventral suction disk was likely an ancestral feature of the snailfishes that has been lost at least three times over their evolution.

### Changes in bone density

**Fig. 7:**
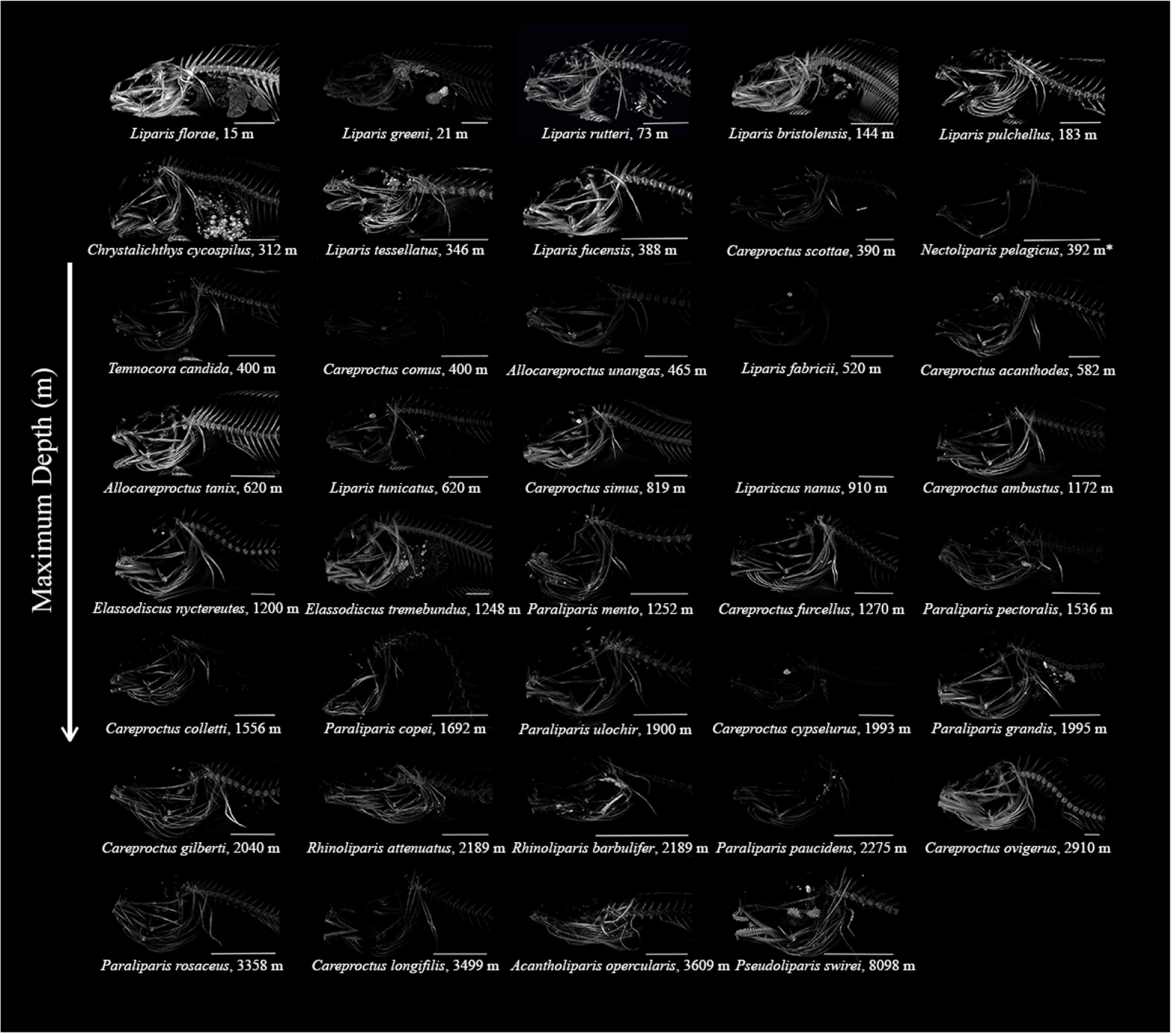
Visualization of bone density with habitat depth across the snailfishes, shown in micro-CT scan images. Brightness settings are standardized throughout the figure, serving as a proxy for bone density. Decreasing density is apparent with increasing maximum habitat depth in the family. Species are grouped by genera in columns, with depth on the y-axis. Scale bars are 1 cm

**Fig. 8:**
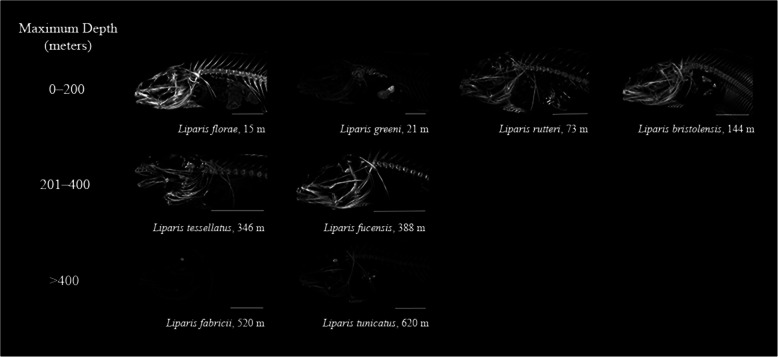
Bone density declines with habitat depth in the genus *Liparis*, shown in micro-CT scan images. Maximum habitat depth ranged from 15 m to 620 m: *Liparis florae*, 15 m, temperate, demersal; *Liparis greeni*, 21 m, temperate, demersal; *Liparis rutteri*, 73 m, temperate, demersal; *Liparis bristolensis*, 144 m, temperate, demersal; *Liparis fucensis*, 388 m, temperate, demersal; *Liparis tessellatus*, 346 m, temperate, demersal; *Liparis fabricii*, 520 m, polar, sometimes pelagic; *Liparis tunicatus*, 620 m, polar, demersal. Brightness, a proxy for bone density, is standardized throughout the figure, with decreasing density apparent with increasing depth in the genus. Scale bars are 1 cm

We examined bone density for five skeletal elements of 38 species from 13 snailfish genera by relating scan brightness to the brightness of known hydroxyapatite standards called phantoms (Fig. 7). Scan brightness, a proxy for bone density, was noticeably lower in deeper-living members within genera, as shown for the genus *Liparis* (Fig. 8). Losses of bone density with increasing habitat depth were visually evident across genera throughout the neurocranium and vertebral column (Fig. 7). *Liparis fucensis,* maximum depth 338 m, had the highest bone densities we measured, while *Liparis fabricii*, maximum depth 520 m, had the lowest (**Supplementary Table** **3**).

In our quantitative analysis, no bones decreased density (% hydroxyapatite) with continuous depth according to phylogenetically-independent modeling at an alpha threshold of 0.05. Density of the vertebra and lower jaw correlated positively with minimum habitat depth (vertebra: df = 38, *p* = 0.0000; lower jaw: df = 38, *p* = 0.0001). Otolith density showed some corresponding decrease with increasing minimum depth at an α threshold of 0.1 (df = 11, *p* = 0.0689), though not for maximum or collection depth. Minimum depth yielded the lowest AIC values for the hypural plate and otolith, indicating the best fit model of the three depth metrics tested (Table 3). For the vertebra and lower jaw, the maximum depth model fit best, while for the ventral suction disk, collection depth resulted in the lowest accompanying AIC values (Table 3). When depth was treated as a categorical variable by ocean zone, depth trends for most bones matched those found in Table 3, with the exception of the vertebra (α = 0.1; PGLS categorical maximum depth: df = 37, coefficient = − 5.3864, *p* = 0.0781; categorical collection depth: df = 37, coefficient = − 9.00328, *p* = 0.076) and otolith (α = 0.1; PGLS categorical maximum depth: df = 10, coefficient = − 21.99286, *p* = 0.0916) which decreased with increasing depth.

**Table 3:** The relationship between depth skeletal element densities according to PGLS significance tests

Density	Minimum Depth				Maximum Depth				Collection Depth			
	*df*	*Coefficient*	*p-value*	*AIC*	*df*	*Coefficient*	*p-value*	*AIC*	*df*	*Coefficient*	*p-value*	*AIC*
Vertebra	38	0.3390429	**0.0000	103.0	37	-0.1893747	0.1389	**73.91**	37	0.0433650	0.7516	117.9
Lower Jaw	38	0.1818685	**0.0001	57.47	37	-0.0336158	0.6948	**45.32**	37	0.0437742	0.5565	72.59
Disk Pterygiophore	20	-0.0706163	0.7052	48.64	20	-0.2065099	0.2467	47.27	19	-0.0884126	0.4296	**47.14**
Hypural Plate	15	-0.0435267	0.4954	**17.39**	15	0.0046979	0.9672	17.95	14	-0.0144411	0.8260	17.67
Otolith	11	-0.0815815	*0.0689	**6.909**	10	-0.1185474	0.5741	8.919	11	0.0596351	0.7592	11.06

Specimen collection depth, species minimum depth, and species maximum depth (in meters) were tested independently. Total degrees of freedom (df) for each test are shown. Significant *p*-values below an alpha threshold of 0.05 are marked with two asterisks and bold type, those below the 0.1 alpha threshold are marked with a single asterisk and bold type. Akaike information criterion (AIC) values are reported for each model. The depth metric with the lowest AIC value for each bone appears in bold type

Individual bones varied in density across the specimens studied. Within each specimen, relative bone density of each structure remained consistent, with otoliths being the densest structures measured (two-way ANOVA vertebra:otolith F_1,9_ = 13.71, *p* = 0.0049; lower jaw:otolith F_1,9_ = 15.72, *p* = 0.00328; disk:otolith F_1,5_ = 7.554, *p* = 0.0404; hypural plate:otolith F_1,4_ = 5.641, *p* = 0.0764). The disk was generally denser than the other bones measured (disk:vertebra F_1,18_ = 81.08, *p* < 0.0001; disk:lower jaw F_1,18_ = 52.71, p < 0.0001; disk:hypural plate F_1,11_ = 128.8, *p* < 0.0001), followed by the lower jaw (lower jaw:vertebra F_1,36_ = 277.1, *p* < 0.0001; lower jaw:hypural plate F_1,14_ = 169.6, *p* < 0.0001), the vertebra (vertebra:hypural plate F_1,14_ = 127.9, *p* < 0.0001), and finally the hypural plate. A few species deviated substantially from this trend. The vertebra and lower jaw of *Liparis tessellatus* were substantially denser than the hypural plate and disk*. Liparis fabricii* had a comparatively low-density vertebra relative to other bones. *Nectoliparis pelagicus*, the only exclusively pelagic species analyzed, had the lowest density otoliths, both overall and relative to its bony skeleton (**Supplementary Table** **1**).

**Fig. 9:**
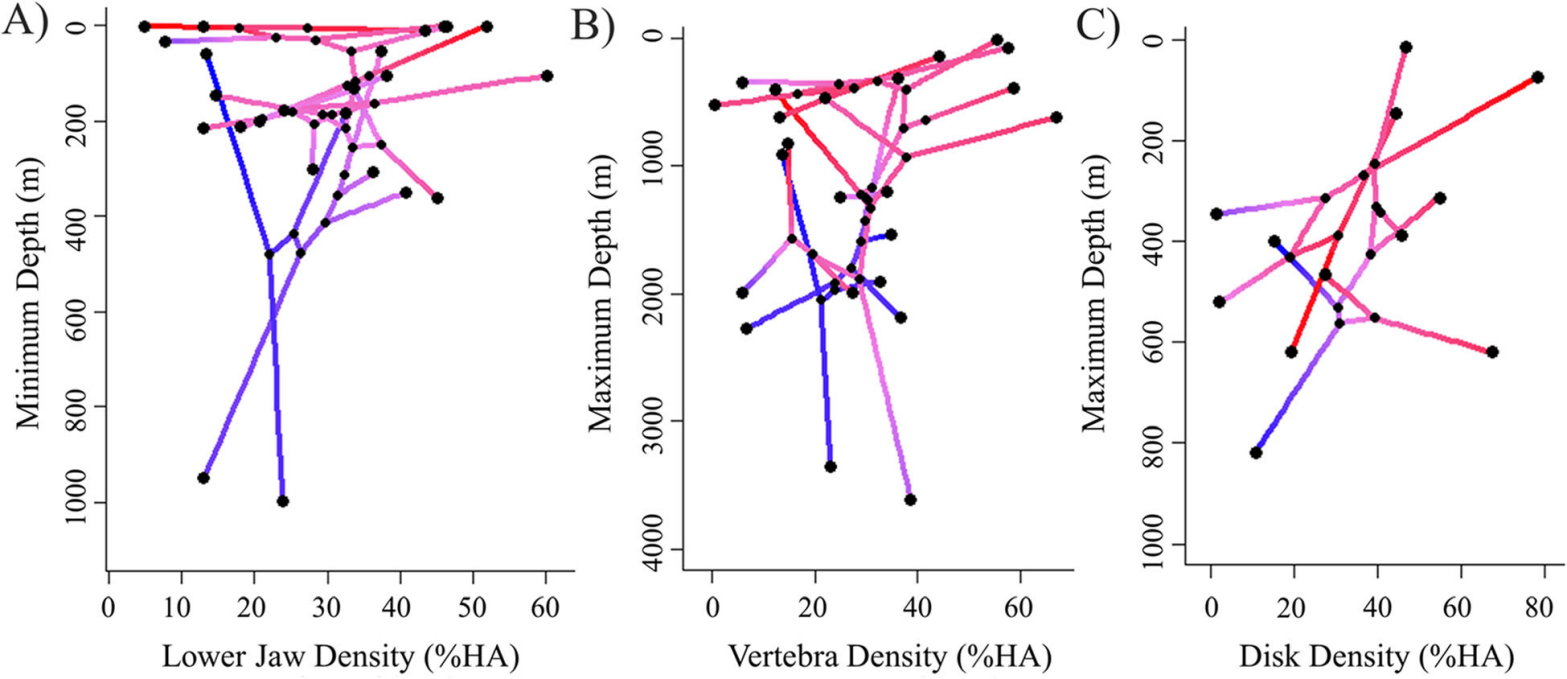
Phylomorphospace visualization of bone density with habitat depth (m), considering phylogeny. Connections between data points indicate phylogenetic relationships. Colors represent standard-length corrected dimensions of related bones (red: longest, blue: shortest). **a** Lower jaw density with minimum depth, color indicates body size-corrected dentary length. **b** Vertebra density with maximum depth, color indicates body size-corrected neurocranium length. **c** Disk density with maximum depth, color indicates body size-corrected maximum disk width

Density and dimension of skeletal structures were often correlated, as shown in phylomorphospace visualizations (Fig. 9). For example, in certain deep-living taxa, lower jaw ossification and reduced dentary length were present, while some low-density, shallow-living taxa showed little reduction in dentary length (Fig. 9**a**). Vertebral density and neurocranium length showed a similar trend, though the relationship was less pronounced (Fig. 9**b**). The polar taxa *Liparis tunicatus* and *Liparis fabricii* displayed lower-density bones than others in the genus, as was also visually evident (Fig. 8). A generalized reduction in suction disk density with increasing habitat depth was accompanied by a decrease in disk width (Fig. 9**c**).

There were also visual differences in density among specimens in bones that were not quantitatively analyzed. The neurocranium especially appeared to exhibit a wide range in density, with deep-water species tending to be less dense compared to intertidal species (Fig. 7), although this trend seems non-linear. While the neurocranium was reduced in density with increasing habitat depth, a crescent of more visibly ossified bones remained—the jaw bones, branchiostegal rays, and pectoral girdle. In analyzing the micro-CT three-dimensional images, we noticed a shift toward more rod-like narrow bones in species found at greater depths. This was especially clear in the maxilla and opercular series. In some species, such as *Paraliparis paucidens* and *Elassodiscus tremebundus*, variations in density in the other jaw bones were apparent, with a plate-like region of low density bone surrounding a rod of higher density bone.

The relationship between bone density and depth was found to be complex and non-linear. Bone density also seemed to be related to other ecological parameters, such as lifestyle and latitude, which are provided for all taxa in **Supplementary Table** **3**, as well as to phylogenetic relationships. There are only a few liparids that are exclusively pelagic, limiting statistical power in comparing bone density across habitats. Qualitatively, however, vertebra and jaw densities appeared to be lower in pelagic species than in demersal taxa. *Nectoliparis pelagicus* had the lowest density otoliths**.** Pelagic species lacked suction disks, so disk density could not be compared across lifestyles. When accounting for phylogeny, latitude was a significant predictor of vertebral density, with polar species having the lowest density vertebrae (PGLS, df = 38, *p* = 0.0029, coefficient = − 2.33052, AIC = 370.6676). Lower jaw density also decreased with increasing latitude (PGLS, df = 38, *p* = 0.0002, coefficient = − 0.81813, AIC = 349.1181). Polar species appeared to have qualitatively lower disk and hypural plate densities. *Liparis fabricii, Liparis tessellatus,* and *Careproctus simus* showed the lowest density suction disks and *Liparis tessellatus* and *Careproctus comus* had the lowest density hypural plates. When polar taxa were excluded from the analysis, vertebral density significantly decreased with minimum habitat depth (PGLS, α = 0.1, df = 36, *p* = 0.0814, AIC = 104.40, Coefficient = − 0.100549).

## Discussion

Using micro-CT scanning, we found evidence for declines in skeletal dimension and loss of pelvic suction disks with increasing habitat depth in the snailfishes. The anecdotal reports of reduced bones in deep-sea fishes were supported in part by the present analysis, but our results reveal complexity in this relationship not previously described and challenge the paradigm that deep-sea fishes have decreasing bone density with increasing habitat depth. In the snailfishes, bone density appears to be related not only to depth, but to lifestyle and habitat. Our qualitative results suggest that pelagic and polar species have lower-density bones, providing an important direction for future study. There are three different routes to skeletal loss —loss of elements, reduction in element size, and reduction in mineralization of elements. We found different lineages that show each of these and some combinations. Together, our results indicate that multiple environmental drivers influence skeletal declines in snailfishes. These drivers include, but are not limited to, increasing hydrostatic pressure. Skeletal reductions in fishes affect multiple physiological functions, including buoyancy, feeding, and locomotion; the implications of which are discussed below.

### Buoyancy

Increasing hydrostatic pressures have been hypothesized to drive bone reductions in deep-sea species. However, our results suggest that the influence of increasing hydrostatic pressure on reducing bone density is both indirect—as a means to maintaining neutral buoyancy or a response to other environmental conditions—rather than a direct effect on bone mineral formation and not the only environmental factor important for bone density. Maintaining neutral or near neutral buoyancies is increasingly difficult for fishes under high hydrostatic pressures of the deep sea, where gas-filled swim bladders are energetically costly to inflate [89, 96]. Fishes have multiple strategies for achieving neutral buoyancy, including large lipid deposits [23, 27], gelatinous tissues [28, 40], and low-density cranial fluids [49]. Skeleton-wide reductions were visible when comparing shallow-living and deep-living taxa of the present study (Fig. 7). Our finding that pelagic taxa seem to have lower density bones than demersal taxa, independent of habitat depth, support the idea that maintaining buoyancy is a strong driver of skeletal reductions. Further, shallower-living polar taxa that lack swim bladders can still achieve neutral and near-neutral buoyancy with reduced skeletal density, such as the notothenioids [26]. We show here that polar liparids also follow this trend. Lower locomotory capabilities and metabolic rates in deep-sea species [24] would also drive increased evolutionary pressure to maintain neutral buoyancy for energetic efficiency.

We observed a clear reduction in ossification and length of the neurocranium in deep-water species. The dorsal portion of the skull was frequently one of the least dense areas observed in deep-sea specimens. A recent study describing the genome of the Mariana snailfish, *Pseudoliparis swirei*, also noted that this species has an incompletely ossified neurocranium or “open skull,” which the authors interpret as a necessary adaptation to survival at high pressure because a “closed skull” would pop under hadal pressures [108]. While our findings and experience with dissections agree with this observation of reduced skeletal density in the bones of the neurocranium, we interpret this result differently. Reduced ossification of the neurocranium is not an adaptation for pressure equalization with the surrounding environment, but rather a means to achieve neutral buoyancy and a result of reduced selective pressures for strong protective skulls in the hadal environment. The logical flaw in the “open skull” hypothesis is that no skull is a completely sealed chamber of bone. The brain sac, within the skull, serves to maintain intracranial pressure in terrestrial vertebrates. In some fishes, these brain sacs are well-ossified (e.g. [44]), but all have openings in the bone for connections to the nervous system. With the contextual CT data from shallow-living liparids presented here, it is clear that snailfishes, like all vertebrates, do not have skulls that are completely sealed with bone, even in shallow-water environments. As another illustration, mackerel are commonly used as bait in hadal camera and trap deployments (e.g. [61]). Although these are shallow-living fishes, their skulls show no pressure-related mechanical damage upon retrieval from 10,000 m. Rather than an adaptation to physiological stress of high pressure, the reduction in bone mineral density of the neurocranium may be possible as a result of the reduced predation pressure on organisms in the deep sea [46], particularly in the case of the hadal snailfishes, which are the top known predators of the hadal zone [41]. It is likely that the majority of the bone is replaced by large areas of cartilage, as in the Antarctic snailfish, *Paraliparis devriesi* [28].

Does hydrostatic pressure have a direct physical effect on bone mineral density? Otoliths are calcium carbonate structures formed passively, in contrast to bones (e.g. [9, 18]), thus serving as a test of the direct pressure effect on bone mineral density. We found that the density of the sagittal otoliths did not decline with increasing collection depth, suggesting that the depth-related density declines seen in other structures are not a direct physical effect of pressure. Interestingly, hadal snailfishes live well below the calcium carbonate compensation depth, where calcium carbonate dissolves faster than it can be formed due to the effects of pressure (e.g. [5]). The Mariana snailfish, *Pseudoliparis swirei*, also showed fully mineralized otoliths (**Supplementary Table** **3**), despite its great habitat depth of 6000–> 8000 m, suggesting differences in the internal environment rather than the surrounding seawater. Our findings that lifestyle and habitat strongly influence bone density also indicate that reductions in bone density are not driven by physical pressure effects on mineralization.

### Feeding

Decreases in bone size were not restricted to the neurocranium as predicted, but rather seen variably in the dentary and suborbital bones. Reductions in bones used for feeding need to be considered within that ecological context. In fishes that have protrusible jaws, a long ascending process allows the premaxilla to slide forward and downward when the jaws are opened (e.g. [109]). This jaw protrusion greatly enhances the forces exerted on prey during suction feeding [48] and moves the opening of the mouth closer to the prey in a behavior termed “jaw ram” [62]. Thus, it is likely important to maintain a long ascending process due to its important role in enabling effective suction feeding, even at great depths. The lack of a decrease in ascending process length with depth could indicate that the ascending process is a more critical portion of the premaxilla bone structure to maintain for effective feeding under the evolutionary pressure for a reduced skeleton.

The maxilla also plays a crucial role in suction feeding by rotating forward to form the bounds of the rounded gape and by applying force to the premaxilla to direct anterior jaw protrusion [25, 31]. The lack of correlation between maxilla length and width and increasing depth indicates the evolutionary importance of maintaining the proportional dimensions of the oral cavity for suction feeding at all depths. The observed prevalence of more rod-shaped bones and visible declines in bone mineral density across the surface of the bone in deep-water specimens might indicate a selective pressure to reduce cross-sectional area of the bone. Thus, the feeding functionality of the bone is maintained while also reducing total bone volume in response to deep-water environmental constraints.

### Locomotion

Reductions in vertebral density with increasing minimum depth of occurrence (polar taxa excluded) may correspond to reduced locomotory capability. This may be in part due to an increase in swimming efficiency and a tendency toward elongation in deep-water species [70]. It is likely that energy savings and metabolism are main drivers that influence adaptation into deep-sea habitats (e.g. [70]). The decrease in metabolic rates with increasing habitat depth is thought not to be a result of only high pressures and low food availability, but rather a decrease in interaction distances between predator and prey with declining light levels [11, 24]. In surface waters, where visual cues dominate predator-prey relationships, these interactions occur over a larger spatial scale. At depth, where mechano- and chemo-sensory signals dominate the sensory landscape, fishes are not under such selective pressures to cover large distances and maintain high metabolic rates. This visual interactions hypothesis seems to be reflected not only in metabolic rates of deep-sea demersal taxa (e.g. [22]), but also in watery muscles (e.g. [21, 112]), and decreased bone density (Present Study). Declining light levels bring decreased selective pressure for robust skeletons and fast swimming, as reflected by the decline in third vertebra densities with increasing habitat depth when excluding polar taxa, although limited to an alpha threshold of 0.1. Hypural plate densities, which would also be indicative of locomotory capabilities, did not significantly decline with increasing habitat depth, although sample sizes for this bone were comparatively small. It is also possible that reductions in pectoral girdle length could be due to the variation in pectoral girdle shapes observed.

We found depth-related declines in the width of the suction disk and loss of the suction disk in some deeper-living taxa. A decrease in wave action and flow rate with increasing depth likely reduces the need for strong suction disks [1]. Predation risk also declines significantly with increasing depth [46], decreasing the need to hide on and among rocks with the help of the suction disk. However, not all deep-living species have lost the ventral suction disk. At least two deep-water snailfishes have been observed using the suction disk in situ*, Careproctus ovigerus* and *C. melanurus* [107], suggesting that despite lower predation risks and current speeds, the suction disk remains useful in some deep-sea groups. There is a strong phylogenetic component to disk reduction and loss (e.g. [82]), which could mask habitat effects in our phylogenetically corrected analysis of bone density. Our ancestral state reconstruction results agree with the predictions of Orr et al. [82], which suggest at least three independent losses of the pelvic suction disk across the evolution of the snailfishes.

### Habitat differences

While habitat depth was found to be a significant predictor of skeletal form across several bones, depth is not the only factor that characterizes a species’ environment. Bone density appeared to be lower in both polar and pelagic taxa, independent of depth. *Liparis fabricii* and *Liparis tessellatus* (e.g. [10]), both Arctic species, showed dramatically low bone densities. Further, *Liparis fabricii* can be pelagic and in our qualitative analyses, we observed that pelagic taxa (e.g. *Lipariscus nanus, Nectoliparis pelagicus*) displayed low-density bones. The differences we observed between bone density in pelagic and demersal taxa were non-trivial. For example, the estimated mean of vertebra and lower jaw density in pelagic taxa were three times lower than the means of demersal species, although this comparison has limited statistical power due to the low number of extant pelagic liparids. Evolutionary history could also constrain bone density. For example, *Lipariscus nanus*, which lives in the ocean’s upper 1000 m, is most closely related to the deeper-dwelling snailfishes of the genus *Paraliparis*. The low-density bone in *L. nanus* may reflect a radiation into shallower water from a deeper population in addition to the species’ pelagic lifestyle. Low-density polar species and the observed depth-related declines in bone density, suggest that temperature could be an important evolutionary driver of bone density, in addition to hydrostatic pressure.

### Depth metrics

**Fig. 10:**
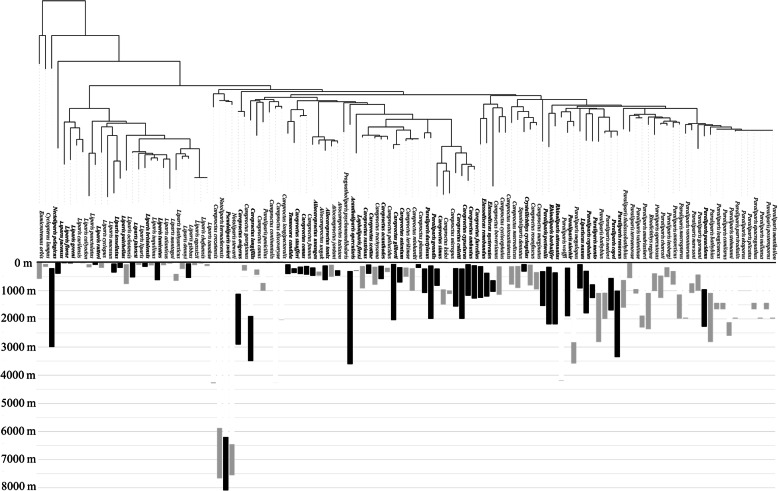
Reproduction of snailfish phylogeny by Orr et al. [82] based on mitochondrial cytochrome *c* oxidase subunit I (COI) sequences, related to depth of occurrence ([10, 50]; Table 4). This phylogenetic tree was visualized using the Interactive Tree of Life (iTOL [60];). Taxa analyzed in this study are shown in bold, with depth ranges in black. Depth ranges for taxa not analyzed in this study appear in gray. Sequences were available for 117 of the 430 described snailfishes, covering all major genera in the family. The maximum depth for *Nectoliparis pelagicus* shown here is believed to be greatly overestimated due to open trawl collections [10] and was not used in statistical analyses in the present study

Our results varied depending on the different depth metrics chosen: collection depth, minimum habitat depth, and maximum habitat depth. Liparid habitat depth ranges can be large (e.g. Figure 10), so it is likely that changes in bone density are gradual and can be masked by overlapping depth ranges when considering just minimum habitat or specimen collection depth. We know also that the habitat depth information presented here is incomplete. Some species are known from only one or two collections. Further, while extremely valuable, databases such as FishBase [33] do not always provide full habitat characterizations for each species or can present erroneous depth values. With missing values and the potential for confounding ranges due to non-closing collection equipment, it is important to use such databases as a starting point, but follow with deeper research into the primary literature and museum archives to confirm ranges (e.g. [85]). Basing our analysis on the most complete information possible and testing multiple habitat depths provided additional insights into depth-related trends.

### Should depth-related trends be linear?

 We do not see significant declines between 0 and 100 m habitat depth (e.g. Figure 8), which is as expected given pressure effects on physiology are negligible at these depths (e.g. [101]). It is also likely that depth-related changes in deep-sea fish skeletons are not linear. Depth in and of itself is not an environmental factor of biological relevance. Indeed, the only environmental driver that changes linearly with depth is hydrostatic pressure (e.g. **Supplementary Fig.** **2**). Our results suggest that other environmental drivers influence skeletal density and dimension, such as declining light levels and changing temperatures. If temperature, light, and nutrient availability drive skeletal evolution, we would expect to see the greatest changes in bones in the first 1000 m from the surface. Most of the species we analyzed live in the 0–1000 m range, so it was not possible to fully explore the nuances of depth-related declines in skeletons in bathyal and abyssal habitats. With additional sampling, particularly in deeper waters near and beyond 4000 where few liparids have been collected (e.g. [37], Fig. 10), this hypothesis could be tested. In addition to increasing depth resolution, future studies should directly test for the effects of varying environmental parameters such as temperature and light and nutrient availability on bone density in fishes.

### Ontogeny and future directions

Ontogeny is also an important consideration in assessing both depth metrics and density results as a whole. Many deep-sea fishes show ontogenetic changes in distribution, moving to different habitat depths throughout their lives, for example downslope migration in *Microstomus pacificus*, Dover sole (e.g. [52]). Compositions of muscle tissue are known to change with these changes in habitat depth [21]. It is possible that bone density and relative dimension also changes with ontogeny. Little is known about snailfish ontogeny [38, 63, 100]. Limited sampling and life history information has precluded full analyses across sizes, ages, and sexes in the snailfishes. Future research should examine bone density across life history stages by focusing on specific genera to better resolve these trends. Our observations here also call for further investigation of bone density in polar taxa and detailed comparisons of skeletal morphology between fishes with pelagic and demersal lifestyles.

## Conclusions

Snailfishes have at least two routes for bone reduction in response to deep-sea environmental conditions: reduction of bone size and loss of unused skeletal elements. Reduction in bone size in the neurocranium, suborbital, dentary, and disk width indicate a complex response to environmental conditions in the deep sea. Bone size was differentially reduced with increasing habitat depth in each functional system (jaw, pelvic suction disk, and neurocranium) to retain specific key functions in feeding and swimming. Bone density of the vertebrae and otolith decreased by ocean zone, while density of the hypural plate, lower jaw, and ventral suction disk did not. These declines could be associated with trends for increased paedomorphy across depth and phylogeny in the snailfishes [26, 63]. Skeletal reduction may be less constrained in fishes because bones have little role in calcium regulation compared to terrestrial vertebrates.

Our visual results support the general idea that bone density decreases in deep-sea fishes, but there is complexity in this trend that contradicts the prevailing paradigm, presumably driven by both physiological and ecological drivers. We found that latitude also significantly predicted bone densities, more so than habitat depth, and qualitative trends in density were apparent with species’ lifestyle (i.e. pelagic vs. demersal). Working within one family (Liparidae) allowed systematic testing of skeletal declines with depth, reducing the influence of phylogeny. We propose that these trends apply across other families under the influence of the same ecological drivers. Future studies should test skeletal reduction across other wide-spread demersal taxa covering broad bathymetric ranges, for example the rattails (Macrouridae), cusk eels (Ophidiidae), eelpouts (Zoarcidae), and cutthroat eels (Synaphobranchidae). The varied ecology and physiology of these other taxa may provide additional insight into the drivers and adaptations of skeletal reductions in deep-sea fishes.

## Methods

### Micro-CT Scanning of Snailfishes

To visualize skeletal morphology and compare relative bone densities of 50 species among 13 genera across the family Liparidae, we used micro-computed tomography (micro-CT), a non-destructive sampling technique (Fig. 10). Specimens spanning collection depths of 0–8000 m were loaned from the University of Washington Burke Museum Ichthyology Collection (UWFC), the University of Hawaiʻi (from J.C. Drazen, *Careproctus longifilis*, [107]), and the Smithsonian Institution’s US National Museum of Natural History (USNM, Table 4). Prior to scanning, specimens were photographed and standard lengths, total lengths, and head lengths were measured in ImageJ (Schneider et al., 2012).

**Table 4:** Specimens in the family Liparidae analyzed for skeletal comparisons in the present study

Genus	Species	Museum ID	SL (mm)	Collection Depth (m)	Minimum Depth (m)	Maximum Depth (m)	Depth Source
*Acantholiparis*	*Acantholiparis opercularis*	UWFC 152662	75.5	1000	300	3609	[10, 65]
*Allocareproctus*	*Allocareproctus kallaion*	UWFC 112244	164.0	441	278	458	[78]
*Allocareproctus*	*Allocareproctus tanix*	UWFC 112294	68.9	158	104	620	[78]
*Allocareproctus*	*Allocareproctus unangas*	UWFC 112308	79.9	387	176	465	UW 113690; [78]
*Careproctus*	*Careproctus acanthodes*	UWFC 117962	116.3	150*	114	582	[80]
*Careproctus*	*Careproctus ambustus*	UWFC 155338	148.5	709	58	1172	[74]
*Careproctus*	*Careproctus bowersianus*	UWFC 153204	130.0	848	629	1032	[10]; UW 113836
*Careproctus*	*Careproctus colletti*	UWFC 118721	179.6	994	200	1556	UW 47284; [47]
*Careproctus*	*Careproctus comus*	UWFC 111841	77.7	303	146	400	UW 113706; [81]
*Careproctus*	*Careproctus cypselurus*	UWFC 156784	107.5	1017	214	1993	[67]
*Careproctus*	*Careproctus faunus*	UWFC 111867	106.3	348	120	422	[81]
*Careproctus*	*Careproctus furcellus*	UWFC 117362	107.1	933	98	1270	[10]
*Careproctus*	*Careproctus gilberti*	UWFC 155468	100.8	348	50	2040	UW 11384;3 [22]
*Careproctus*	*Careproctus longifilis*	T395#7	77.3	–	1900	3499	[10, 107]
*Careproctus*	*Careproctus ostentum*	UWFC 119726	105.0	332	165	700	[10]
*Careproctus*	*Careproctus ovigerus*	UWFC 118518	190.5	1109	1109	2910	[76]
*Careproctus*	*Careproctus phasma*	UWFC 117915	141.0	76	57	184	[80]
*Careproctus*	*Careproctus scottae*	UWFC 028329	145.0	203	71	390	[80]
*Careproctus*	*Careproctus simus*	UWFC 156797	147.0	455	213	819	UW 118689; UW 119035
*Careproctus*	*Careproctus staufferi*	UWFC 155804	81.6	256	205	366	[75]
*Crystallichthys*	*Crystallichthys cyclospilus*	UWFC 117257	126.7	120	54	312	[10]; UW 154865
*Elassodiscus*	*Elassodiscus nyctereutes*	UWFC 119414	181.5	616	362	1200	[54]
*Elassodiscus*	*Elassodiscus tremebundus*	UWFC 119576	155.8	700	130	1248	[10]
*Liparis*	*Liparis bristolensis*	UWFC 113161	62.3	10	10	144	[10]; UW 152009
*Liparis*	*Liparis fabricii*	UWFC 118038	89.9	28	0	520	[66]
*Liparis*	*Liparis florae*	UWFC 040065	87.7	0	0	15	[86]
*Liparis*	*Liparis fucensis*	UWFC 049751	54.5	45	0	388	[86]
*Liparis*	*Liparis gibbus*	UWFC 155347	128.8	38	30	540	[10]
*Liparis*	*Liparis greeni*	UWFC 010441	105.4	0	0	21	[86]
*Liparis*	*Liparis pulchellus*	UWFC 118508	105.3	33	9	183	[86]
*Liparis*	*Liparis rutteri*	UWFC 155451	66.9	38	0	73	[86]
*Liparis*	*Liparis tessellatus*	UWFC 042613	110.2	34	34	346	[84]
*Liparis*	*Liparis tunicatus*	UWFC 153017	77.9	43.8	0	620	[13]
*Lipariscus*	*Lipariscus nanus*	UWFC 154989	65.4	477	58	910	[10]
*Lopholiparis*	*Lopholiparis flerxi*	UWFC 047868	32.5	278	121	285	[65, 77]
*Nectoliparis*	*Nectoliparis pelagicus*	UWFC 155729	54.2	392	200	–	[10]
*Paraliparis*	*Paraliparis cephalus*	UWFC 047190	70.0	622	294	1799	[10]
*Paraliparis*	*Paraliparis copei*	USNM 186151	110.3	1463	548	1692	[10]
*Paraliparis*	*Paraliparis dactylosus*	UWFC 118640	90.0	869	212	1073	UW 117718; UW 157764
*Paraliparis*	*Paraliparis grandis*	UWFC 115690	102.3	834	105	1995	[10]
*Paraliparis*	*Paraliparis holomelas*	UWFC 153155	83.0	188	55	2972	[8]
*Paraliparis*	*Paraliparis mento*	UWFC 151861	83.9	776	776	1253	[10]
*Paraliparis*	*Paraliparis paucidens*	UWFC 115461	87.5	1018	950	2275	[79]
*Paraliparis*	*Paraliparis pectoralis*	UWFC 152660	81.4	950	308	1536	UW 117778; [10]
*Paraliparis*	*Paraliparis rosaceus*	UWFC 115465	104.3	999	999	3358	[10]
*Paraliparis*	*Paraliparis ulochir*	UWFC 153044	124.7	1018	182	1900	[65, 105]
*Pseudoliparis*	*Pseudoliparis swirei*	USNM 438975	104.0	7949	6198	8098	[36]
*Rhinoliparis*	*Rhinoliparis attenuatus*	UWFC 117365	118.2	1018	350	2189	[10]
*Rhinoliparis*	*Rhinoliparis barbulifer*	UWFC 150806	69.6	610	149	2189	UW 157184; [10]
*Temnocora*	*Temnocora candida*	UWFC 153162	71.6	238	64	400	[67]

Standard lengths (SL) are shown in mm, depth in meters (m). References for species' depth ranges are listed (min depth; max depth). Some museum specimens from the UWFC extend the published depth ranges for these species; these are noted as sources. All are museum specimens except *Careproctus longifilis*, whose collection is described by [107]. Collection depth for *Careproctus acanthodes* represents the collection lot and is considered an estimate. UW 154989 (*Lipariscus nanus*) and UW 155729 (*Nectoliparis pelagicus*) are pelagic species that were collected with non-closing benthic trawls

Specimens were scanned in batches (1–12 individuals) in a Bruker SkyScan 1173 at 65 kV and 123 μA with a 1 mm aluminum filter to reduce beam hardening (e.g. [6]) at the Karel F. Liem Bioimaging Center, Friday Harbor Laboratories, University of Washington. Scan resolution (voxel size) ranged from 14.9 to 35.5 μm, depending on the size of the fish. Known-density standards of 25 and 75% hydroxyapatite called phantoms either 7.5 or 10.5 mm in diameter were included in each scan to relate relative brightness to density. The inclusion of the same phantoms of similar thickness to the fishes in each individual scan allowed for a reduction in beam hardening to minimize inconsistency in X-ray attenuation. Scans were reconstructed using NRecon (Bruker, 2005–11) under standard procedure for optimal x/y alignment, ring artifact reduction, beam hardening correction, and post-alignment. The reconstructed scans were further segmented in DataViewer (Bruker, 2004–11). All scan reconstructions from this study are freely available for download on MorphoSource (Duke University).

### Quantification of skeletal morphology and density

To test the first hypothesis—that dimensions of select skeletal structures are reduced with increasing habitat in fishes—we measured linear dimensions of bone regions in Horos [17]. We chose 12 dimensions and four bone regions to capture functionally relevant structural variation among species and size. Jaw measurements were as follows: maxilla length, maxilla width, premaxilla length, length of the ascending process of the premaxilla, dentary length (dorsal process), dentary length (ventral process), and anguloarticular length. Skull measurements included neurocranium length and suborbital length and width. On the ventral side of the fish, we measured the maximum width of the pelvic suction disk. Lastly, we measured the length of the pectoral girdle, the linear distance from the dorsal end of the scapula to the ventral end of the coracoid. These bones are illustrated in Fig. 1**.**

To test the second hypothesis—that skeletal structures that are not selected for in deep-sea habitats would be lost with increasing depth—we examined scans for missing skeletal elements. Following identification of specific lost structures, ancestral state reconstruction using a maximum likelihood framework was used to estimate the number of independent losses of certain skeletal elements among liparids. With the *phytools* package [94] in the statistical programming platform R [91], we obtained marginal ancestral state estimates and the phylANOVA function to test for an effect of depth in predicting structure loss.

To test the third hypothesis—that bone density decreases in fishes with increasing habitat depth—we used the 3D processing software Amira [103] to measure mean pixel brightness of individual bones as a proxy for bone density. For data visualization in Amira, colormaps were standardized across scans using a standard curve to achieve a range representing 10–90% hydroxyapatite. To reduce uncertainty at the variable ends of the brightness spectrum, all pixel values beyond the edges of this range became minimum (black) or maximum (white) values. This standardized the color scale to allow scans to be compared visually, with darker regions corresponding to lower X-ray absorption, or density, and lighter regions corresponding to higher absorption or density (e.g. [95]). These brightnesses were compared to known-density phantoms and converted to percent hydroxyapatite. These results are a proxy for bone density, rather than a direct calculation of density, and are treated as relative and purely for purposes of comparing taxa here. We compared relative densities of the third vertebra, lower jaw, first left disk pterygiophore, hypural plate, and sagittal otoliths (Fig. 2). The third vertebra was chosen for consistent comparison between specimens. Vertebral density varied visually across the fish, with posterior-most caudal vertebrae appearing less bright than anterior vertebrae. The first and second vertebrae were sometimes fused, while the third was markedly distinct from the neurocranium. Brightness data were not available for all five bones in each specimen. Due to otolith extraction upon collection, phylogenetic loss of suction disks, and loss of specimen tails, some bones could not be measured. Degrees of freedom for each relationship are shown in Table 3 and reflect sample numbers available.

### Preservation effects

Upon collection, liparids are preserved in a standard solution of 4% buffered formaldehyde, then eventually transferred to 70% ethanol or 50% isopropanol for analysis and long-term storage. If the formalin is unbuffered, the acidic solution can dissolve hard structures in the specimen. Unfortunately, complete records of preservation and the amount of time specimens spent in different preservatives were not available for all museum specimens analyzed here. To test for potential confounding effects of preservation on our bone density measurements, we analyzed the relationship between collection year and pixel brightness. There was no significant relationship between depth and collection year (one-way ANOVA; collection depth: F_1,35_ = 1.268, *p* = 0.268; minimum depth: F_1,36_ = 0.786, *p* = 0.381; maximum depth: F_1,35_ = 3.276, *p* = 0.0789). We found no negative trend between collection year and bone density for almost all bones (one-way ANOVA, third vertebra: F_1,37_ = 3.448, *p* = 0.0713; first disk pterygiophore: F_1,19_ = 0.701, *p* = 0.413; dentary: F_1,37_ = 4.417, *p* = 0.0424*; hypural plate: F_1,13_ = 0.305, *p* = 0.413; otolith: F_1,9_ = 4.197, *p* = 0.0708). There was a correlation between collection year and dentary density, significant at the α = 0.05 threshold, although it is unclear what is causing this decline. Bones in the same specimen of similar rod-like shapes, such as the disk pterygiophore did not correlate in density with collection year. We conclude that preservation did not significantly influence our findings presented below.

### Data analysis

Statistical analyses were conducted in R [91]. To control for the effects of covariation in closely related species [30], phylogenetic generalized least squares (PGLS) models were used to investigate the relationship between habitat depth on bone dimension and density using the comparative method packages *ape* [83], *PHYLOGR* [20], *nlme* [87], and *phytools* [94]. We corrected all morphological data to specimen standard length by computing residuals of phylogenetic generalized least squares models using the function “phly.resid” in the phytools package. We reconstructed phylogenetic relationships of liparid species in our study according to the genetic tree presented by Orr et al. [82]. Cytochrome c oxidase subunit I (COI) sequences were aligned using MUSCLE [29], and a maximum likelihood phylogeny was prepared using RAxML [104] via the CIPRES Science Gateway [68]. Full details on the sequences and phylogeny construction are presented in Orr et al. [82]. We then pruned the phylogeny to only include species in our study for analysis. The ancestral state reconstruction was created using the function *simmap* with 1000 simulations and the ARD model [4, 94].

Deep-sea fishes, including snailfishes, can inhabit broad depth ranges throughout their lifetimes, on the order of thousands of meters (e.g. [10, 52, 88]; Fig. 10). As such, it can be difficult to capture the full habitat depth range of a fish with one metric. To test the effects of habitat depth on skeletal dimensions and bone density, we conducted our statistical analyses using collection depth of individual study specimens, as well as minimum and maximum depth for the species from the literature. Sources for each of these depth ranges are listed in Table 4. This combination of metrics has been an effective way to test depth-related biological parameters in fishes in previous studies (e.g. [22]). To test for broad categorical trends, we binned depths roughly by ocean zone (0 to 10 m, 10 to 200 m, 200 to 1000 m, 1000 to 3000 m, 3000 to 6000 m, and 6000 to 11,000 m), in addition to treating depth as a continuous variable. Intertidal collections from 0 m were corrected to 0.1 m for log transformations. To explore the effects of lifestyle on bone density, taxa were classified as either pelagic or demersal according to the literature (e.g. [10]). Comparisons between pelagic and demersal taxa are treated as qualitative throughout the study because there are very few extant pelagic liparids, limiting statistical power (**Supplementary Table** **3**). We also investigated latitudinal density variation according to the latitude of the species holotype as found in Eschmeyer’s Catalog of Fishes [32]. Statistical results from each of the three depth metrics and latitude are reported and discussed. For each PGLS model, a coefficient and *p*-value are reported with α significance levels set at 0.05 and 0.1. The α threshold of 0.1 increases the risk of Type I error. We report both significance thresholds to most thoroughly examine the dataset and discuss limitations of these conclusions throughout the manuscript. Akaike Information Criterion (AIC) values were used to compare PGLS models built with different depth metrics.

## Supplementary Information

**Additional file 1: Supplementary Figure 1.** Example micro-CT scan of the shallow-living species *Liparis miostomus* (UW #041391).

**Additional file 2: Supplementary Figure 2.** General environmental conditions with increasing habitat depth. Data from the Hawaii Ocean Time Series at Station ALOHA from 1988 to 2019 (Pacific Ocean, 22°45′N, 158°W). Depth profiles vary across latitude, longitude, ocean basin, and season. Data obtained via the Hawaii Ocean Time-series HOT-DOGS application University of Hawaiʻi at Mānoa. National Science Foundation Award #1756517. Light levels decline exponentially with increasing habitat depth, with depths below ~100 m lacking enough light for photosynthesis and depths below ~1000 m having no downwelling sunlight. This figure is meant to illustrate general trends in environmental factors with depth and is not an exhaustive representation of snailfish habitat conditions.

**Additional file 3: Supplementary Table 1.** Linear measurements of liparid species analyzed in the present study. Species and maximum habitat depth in meters (from the literature) are shown (see Table 4 for sources). Dentary dorsal fork length (dfl) and ventral fork length (vfl) are shown. All measurements were taken from preserved museum specimens in micro-CT and are shown in millimeters (mm).

**Additional file 4: Supplementary Table 2.** Relationship between calculated phylogenetic residuals and habitat depth in snailfishes, according to a two-way ANOVA. Degrees of freedom (df) and F-statistic (F) for each test are shown. Significant *p*-values below an alpha threshold of 0.05 are marked with two asterisks and bold type, those below the 0.1 alpha threshold are marked with a single asterisk and bold type.

**Additional file 5: Supplementary Table 3.** Proximate bone density (related to percent hydroxyapatite, %HA) for five important bones in the snailfishes. Species and maximum habitat depth in meters (from the literature) are shown (see Table 4 for sources). Vertebra refers to the third vertebra from the dorsal side of the fish, suction disk to the first left disk pterygiophore from the anterior side of the fish, and otoliths refers to the sagittal otoliths. Some elements were missing in scans and were not possible to measure.

## Data Availability

All CT scans from the present study are available open access on MorphoSource. Length, density measurements, and scan renderings are presented in the manuscript and supplementary materials.

## Abbreviations

AIC: Akaike information criterion
ANOVA: Analysis of Variance
Micro-CT: Micro-computed tomography
SL: Standard length
TMAO: Trimethylamine-N-oxide
UWFC: University of Washington Burke Museum Ichthyology Collection
USNM: Smithsonian Institution’s United States National Museum of Natural History
