# Supplementary figures and images for: Habitat influences skeletal morphology and density in the snailfishes (family Liparidae)
Source: Front Zool. 2021 Apr 16;18:16. doi: 10.1186/s12983-021-00399-9 (PMC8052763; doi:10.1186/s12983-021-00399-9)

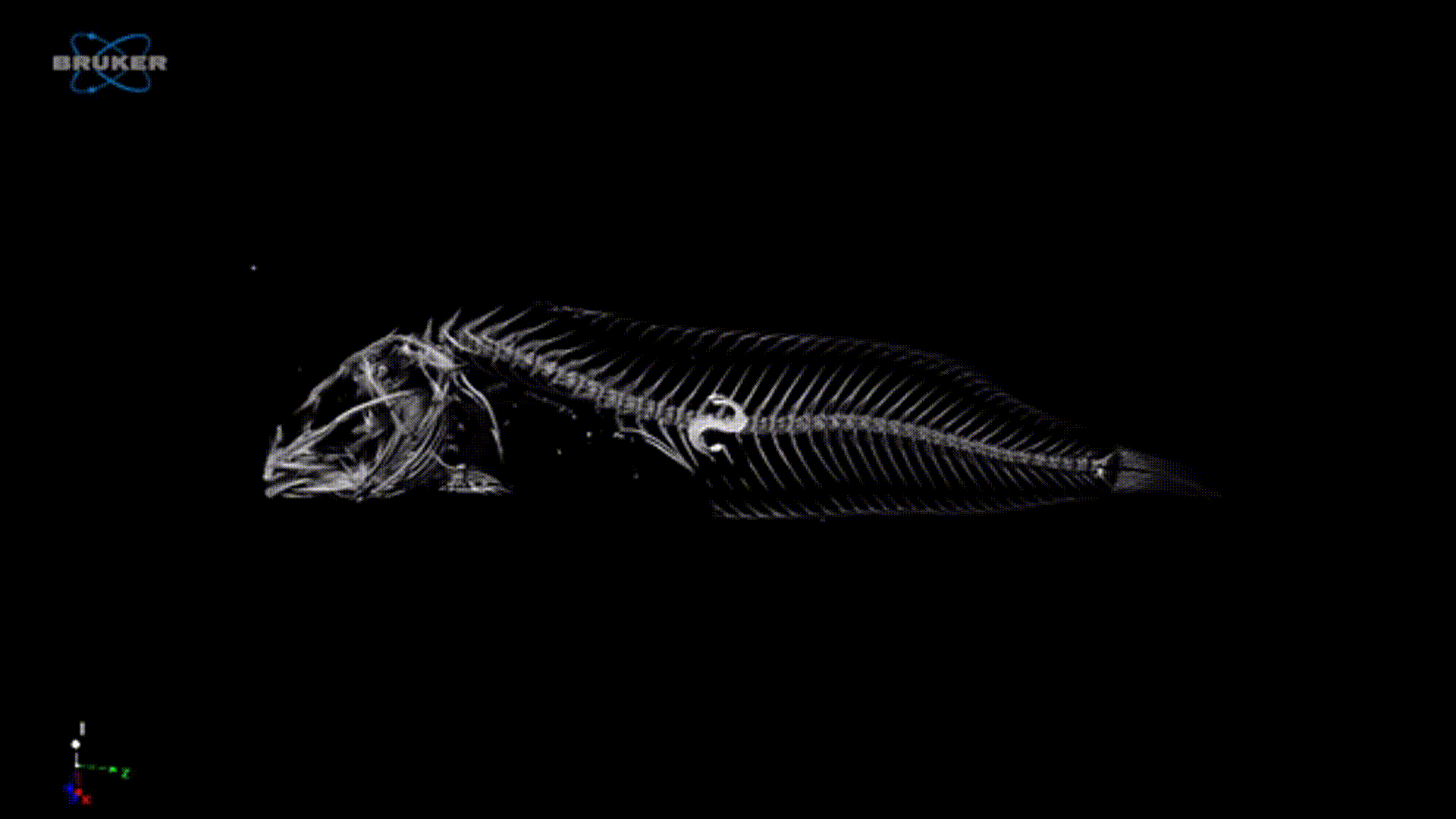

Supplement: Supplementary file 1 — Additional file 1: Supplementary Figure 1. Example micro-CT scan of the shallow-living species Liparis miostomus (UW #041391). [file 12983_2021_399_MOESM1_ESM.gif]

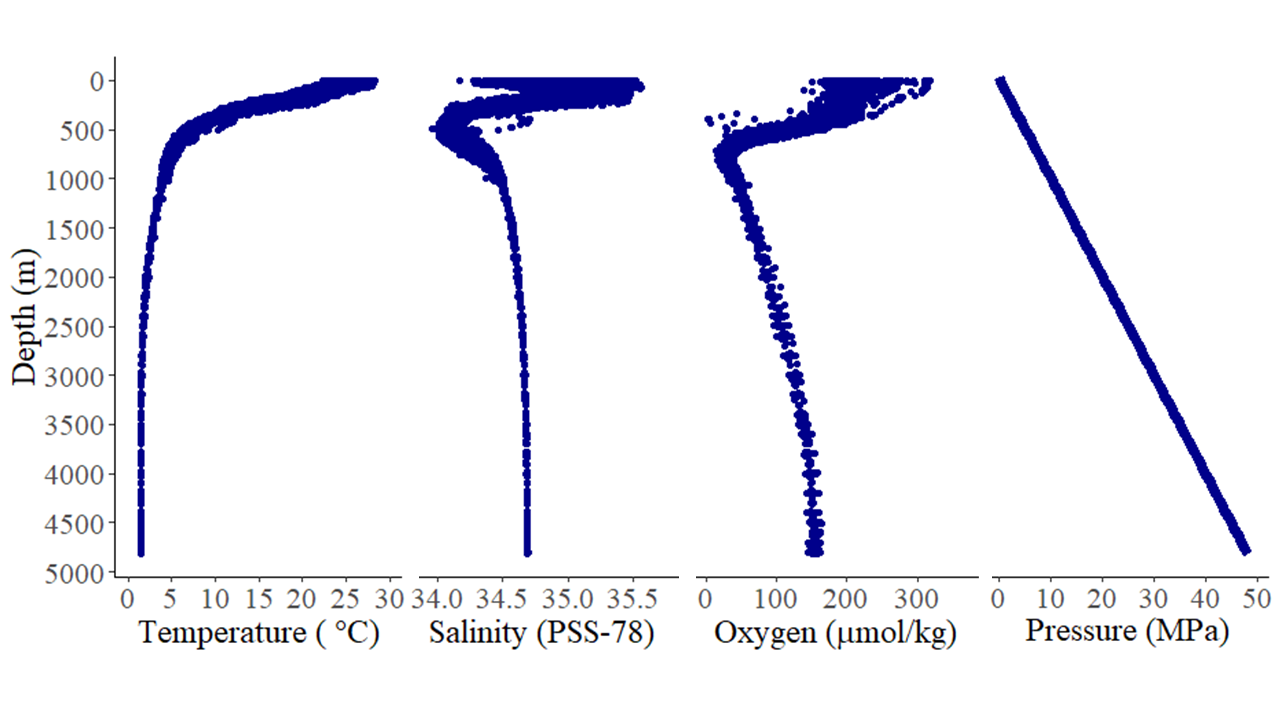

Supplement: Supplementary file 2 — Additional file 2: Supplementary Figure 2. General environmental conditions with increasing habitat depth. Data from the Hawaii Ocean Time Series at Station ALOHA from 1988 to 2019 (Pacific Ocean, 22°45′N, 158°W). Depth profiles vary across latitude, longitude, ocean basin, and season. Data obtained via the Hawaii Ocean Time-series HOT-DOGS application University of Hawaiʻi at Mānoa. National Science Foundation Award #1756517. Light levels decline exponentially with increasing habitat depth, with depths below ~100 m lacking enough light for photosynthesis and depths below ~1000 m having no downwelling sunlight. This figure is meant to illustrate general trends in environmental factors with depth and is not an exhaustive representation of snailfish habitat conditions. [file 12983_2021_399_MOESM2_ESM.tif]
